# Epidemiology of unplanned out-of-hospital births attended by paramedics

**DOI:** 10.1186/s12884-017-1638-4

**Published:** 2018-01-08

**Authors:** Gayle McLelland, Lisa McKenna, Amee Morgans, Karen Smith

**Affiliations:** 1 School of Nursing and Midwifery, Monash University, PO Box 527, Frankston, 3199 Australia; 2 School of Nursing and Midwifery, La Trobe University, Bundoora, VIC 3083 Australia; 3 Department of Primary Health Care, Monash University, Clayton, VIC 3168 Australia; 4 Ambulance Victoria, Blackburn North, VIC 3130 Australia; 5 Department of Epidemiology and Preventative Medicine, Monash University, Clayton, VIC 3168 Australia

**Keywords:** Out of hospital births, Birth before arrival, Childbirth, Ambulance, Paramedic, Prehospital, Emergency care

## Abstract

**Background:**

Over the previous two decades the incidence and number of unplanned out of hospital births Victoria has increased. As the only out of hospital emergency care providers in Victoria, paramedics would provide care for women having birth emergencies in the community. However, there is a lack of research about the involvement of paramedics provide for these women and their newborns. This research reports the clinical profile of a 1-year sample caseload of births attended by a state-wide ambulance service in Australia.

**Methods:**

Retrospective data previously collected via Victorian Ambulance Clinical Information System (VACIS ®) an in-field electronic patient care record was provided by Ambulance Victoria. Cases were identified via a comprehensive filter, and analysed using SPSS version 19.

**Results:**

Over a 12-month period paramedics attended 324 out-of-hospital births including 190 before paramedics’ arrival. Most (88.3%) were uncomplicated precipitous term births. However, paramedics documented various obstetric complications including postpartum haemorrhage, breech, cord prolapse, prematurity and neonatal death. Furthermore, nearly one fifth (16.7%) of the women had medical histories that had potential to complicate their clinical management, including taking illicit or prescription drugs. Mothers were more likely to be multiparas. Births were more likely to occur between 2200 and 0600 h. Paramedics performed a range of interventions for both mothers and babies.

**Conclusions:**

Paramedics provided emergency care for prehospital out-of-hospital births. Although most were precipitous uneventful births at term, paramedics used complex obstetric assessment and clinical skills. These findings have implications for paramedic clinical practice and education around management of unplanned out of hospital births.

## Background

The unpredictable nature of childbirth occasionally results in unplanned ‘out of hospital’ (OOH) births in the pre-hospital setting without medical care. Unplanned OOH births and ‘birth before arrival’ (BBA) are nomenclatures for a birth that occurs before arrival at hospital, or before arrival of the pre-organised midwife at a planned homebirth [1, 2]. OOH births usually occur outside an appropriate health facility [2–5] regardless of whether a midwife or medical professional is present. The definition is often extended to included births occurring in hospitals in inappropriate locations, such as emergency departments, or health facilities without obstetric facilities [1, 6, 7].

Following OOH births there are increased adverse outcomes reported for both mother and baby. Babies born at term, which would otherwise be considered low risk, have substantially greater chance of requiring admission to special care or neonatal intensive care units than similar in-hospital births [4]. All babies born in an unplanned setting, regardless of gestation, have increased risk of hypothermia [1, 8], hypoglycaemia [4, 9] and jaundice [9] resulting in more risk of admission to special care nurseries or neonatal intensive care units [4]. Similarly, following OOH births mothers also have poorer outcomes than their in-hospital counterparts, due to perineal tears and retained placentas increasing the risk of primary postpartum haemorrhage (PPH) [10]. As maternal and neonatal outcomes following planned home births are considerably better than after OOH births [11], it is prudent to investigate them separately.

Coinciding with the closure of maternity services across Victoria, Australia, over the previous three decades (37 since 1997 and 88 since 1983) [12], there has been a steady increase in the number and incidence of OOH births [3]. Although the specific reason for rising incidence of OOH births in Victoria has not been investigated, a direct relationship between this increase and the closure of maternity services has been noted [7, 13]. Little is known about the pre-hospital emergency care these mothers and babies receive.

Victorian paramedics provide pre-hospital emergency medical care for the state, including attending OOH births, hence potentially being the first health professionals providing care for these women and babies. Whilst no Australian studies have been performed investigating the incidence of OOH births attended by paramedics, or the clinical profile of these cases, a recent review of international literature found that paramedics were called to attend between 28 to 89% of all OOH births and were present for 28 to 65% of these births [10]. This study adds to a recent publication that reported upon Victorian paramedics’ transportation of women in labour [14].

The primary aim of this study was to examine the clinical profile of a 1-year state-wide sample of births attended by paramedics including the complications they encountered and the management they performed.

## Method

### Ethics approval

The study was approved by Monash University Human Research Ethics Committee. As this study reports upon data collected during paramedics’ management individual patient consent was not possible, however approval was received from the Research Committee of Ambulance Victoria.

### Study setting

With a land mass of 227,416 km^2^ [15], Victoria has a population of 5 million people. Approximately 80% of the population live in the greater Melbourne metropolitan area covering nearly 2250 km^2^, with the majority of the remainder in regional centres and a small proportion in rural areas. Similarly, more than two thirds of the maternity services are located in the greater metropolitan Melbourne area, with most of the remaining in larger regional areas and very few birthing facilities in smaller rural areas [16]. In the first decade of this century, the number of births in Victoria increased from 61,569 births [17] in 2000 to 72,914 births in 2010 [18].

### Data collection

**Fig. 1:**
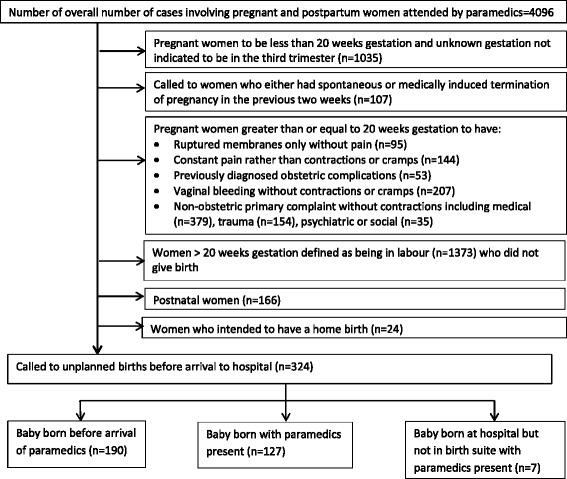
Selection process for inclusion in analysis of paramedics’ attendance at women having a BBA

Victorian paramedics document their management of emergency pre-hospital cases on an in-field electronic patient care clinical information through the Victorian Ambulance Clinical Information System (VACIS ®) [19] that records patients’ demographic data clinical information and paramedics’ management. Using an electronic data filter, retrospective data collected by paramedics between January 1st and December 31st, 2009, cases involving pregnancy, labour and birth were extracted from Ambulance Victoria’s Clinical Data Warehouse which stored the clinical data. The parameters used to select cases for this study were those where the primary presenting problem was ‘childbirth’ or ‘labour’; or the pre-existing condition was ‘pregnancy’; ‘gravida’; ‘para’; or the secondary survey was ‘birth’; ‘childbirth’; or the procedures listed indicated childbirth had occurred e.g. ‘cord cut’, ‘placenta’ or APGAR scores were recorded. As paramedics’ documentation of the cases involving maternity related cases proved unsystematic, the final dataset was reviewed manually to confirm that all cases were women having babies and these were systematically recoded for data analysis. During the recoding of the data it was found that there was substantial missing data however it was decided we would analyse the data that was recorded. Although the final database had 4096 maternity rated cases, there were 324 women who were considered to have births in either unplanned locations or without the intended birth attendants (Fig. 1).

Data extraction included all demographic, operational, clinical data and free text case notes. Inclusion criteria were cases where paramedics had documented the women planned to be in hospital for the births of their babies or those women who intended to birth at home but their babies were born before the arrival of the organised midwife. Exclusion criteria were cases where paramedics attended women who were in labour but did not birth (these were analysed as a separate study), women during pregnancy prior to labour, women who birthed a fetus at less than 20 weeks gestation. As their outcomes are usually much better [11], women who planned a home birth with the midwife present were also excluded. Conversely, cases involving women who intended a planned home but the midwife was not present were included.

### Data analysis

Data were coded to identify variables of interest and descriptive statistical analysis was performed using Statistical Package for Social Sciences (SPSS) version 19. As most of the continuous variables were not normally distributed, they were analysed using both means with standard deviations and medians with interquartile ranges.

## Results

Of the 4096 maternity related cases attended by paramedics in the study period, paramedics were called to more than 2000 women under the call event type of “childbirth/imminent birth”. However, only 324 cases resulted in women who had OOH births including seven babies that were born at hospital but before arrival at the birth suite. Other than APGAR scores, paramedics were not prompted by VACIS to obtain specific pregnancy information including gestation, numbers of previous pregnancies and births leading to incomplete documentation for numerous cases. Additionally, paramedics entered specific obstetric documentation into several different fields including pre-existing conditions, secondary survey and free text resulting in unsystematic documentation requiring substantial recoding. Individual case records of the care for mother and baby were completed on 194 occasions. For the remaining 130 births attended by paramedics, documentation of the care of both mother and baby was on the same record.

### Demographic trends

Demographic data are presented in Table 1. Maternal age ranged from 16 to 44 years (mean age of 29.1 years). Although the majority of women were multigravidas (mean gravida 3.15), this was the first pregnancy in 24 cases. Paramedics noted that at least 45 (13.9%) women had sought medical assistance or advice from the hospital they were booked into earlier in their labours. Nine women (2.8%) attended were documented by paramedics as non-English speaking but this could be underreported as paramedics generally only collect this information when it impacts patient management.

**Table 1:** Maternal demographic data for BBAs

	N	Min	Max	Mean	Median	Q_1_	Q_3_	IQR	SD
Age (years)	313	16	44	29.99	30.0	26.0	34.3	8.3	5.81
Gestation (weeks)	260	20	42	38.4	39.6	38.0	40.0	2.0	3.59
Gravida	253	1	13	3.14	3.0	2.0	4.0	2.0	1.73
Para	254	1	9	2.72	2.0	2.0	4.0	2.0	1.51
SEIFA	303	1	10	5	5	3.0	7.0	4	2.79
^a^Scene time	319	0:1:00	1:41:00	0:19:17	0:17:00	0:11:00	0:24:00	0:13:00	0:13:06
^a^Transport time	319	0:2:00	2:58:00	0:22:03	0:20:00	0:12:00	0:28:15	0:16:15	0:22:03
^a^Patient time	319	0:11:00	3:06:00	0:42:20	0:39:00	0:30:00	0:51:00	0:21:00	0:42:20

^a^Transport and scene times are in hour:minute:second (hour:min:sec)

When classified according to the Australian Standard Geographical Classification - Remoteness Area (ASGC-RA) [15], nearly three quarters of the births (74.1%, *n* = 240) occurred in major cities, nearly one quarter were in inner regional (23.1%, *n* = 75) and 2.8% (9) were in outer regional areas. Paramedics documented residential suburb for 303 women and this was rated against the Socio-Economic Indexes for Areas (SEIFA) [20]. Marginally, there was increased chance that women experiencing an OOH birth were from lower socioeconomic areas with nearly 60% (*n* = 178) of women in suburbs rated five deciles and below. Time of day trends were noted with 72.5% of births occurring outside business hours, with 50.2% occurring overnight between 2200 h and 0800 h.

The time from arrival of paramedics to the woman on scene until their departure to hospital varied from 1 min to 1 h 40 min, however the mean on-scene time was 19 min 17 s (SD 13 min 6 s) (Table 1). The majority of women (*n* = 323, 99.7%) were transported to hospital. Although maximum transport time was nearly 3 h, mean transport time was 22 min and 3 s. The mean total time paramedics spent with the women was 42 min and 20 s but the maximum total patient time was 3 h and 6 min.

### Clinical profile

The majority of OOH births (88.3%, *n* = 227) occurred at term with an overall mean gestation of 38.3 weeks. There was a small proportion of babies (11%, *n* = 29) born at less than 36 weeks gestation. This includes 11 (4.3%) who were very premature, between 24 and 32 weeks gestation.

Initial and final APGAR scores were performed on 235 and 180 babies respectively. When paramedics were in attendance at the baby’s birth, initial APGAR scores were assessed at 1 min after birth. However, when the baby was born before paramedics arrived, the initial APGAR score was performed upon arrival. The final APGAR score was performed before arrival at hospital. Whilst a small number of babies had initial (5.6%, *n* = 13) and final APGAR scores (1.7%, *n* = 3) equal to or less than four, the majority had initial (77%, *n* = 181) and final APGAR scores (93.3%, *n* = 168) equal to or over eight. Mean initial and final APGAR scores were 8.25 and 9.25 respectively. However, for babies who were born before the arrival of paramedics, the initial APGAR score was performed after 1 min so the 1 min APGAR score could have been lower.

Almost two thirds of babies were born prior to paramedic arrival (58.6%, *n* = 190) (Fig. 1). Paramedics assisted 39.2% (*n* = 127) births at the initial scene or en route to hospital. The final 2.2% (*n* = 7) birthed at hospital but before reaching the birthing suite (including the emergency department, corridor, elevator and car park). For OOH births occurring before paramedics’ arrival, other people present were recorded in 117 (61.9%) of cases. The most likely birth attendant for just over half the women was the partner/husband (*n* = 64), followed by another family member (*n* = 21) and neighbour or friend (*n* = 4). One woman was assisted by her local doctor who was not the intended birth attendant and another by a police officer. Two of the women intended home births but the babies were born before the midwives’ arrival. Of concern, nearly 10 % of women (*n* = 26) birthed alone. There were at least seven women encountered who had received no antenatal care with no hospital booked including four (2.1%) with unknown pregnancies and one teenager (0.3%) who had not informed her parents. All four women with unknown pregnancies birthed before paramedics’ arrival.

Third stage data was obtained from the free text and ‘procedure performed’ fields and was reported in 81.2% (*n* = 263) of cases. Of the cases where the third stage was documented, the placenta spontaneously birthed in the presence of paramedics in 36.9% (*n* = 97) and remained in utero for 63.1% (*n* = 166) of women.

### Complications encountered by paramedics at OOH births

Most of the OOH births encountered by paramedics were uneventful, however 31 (9.6%) involved obstetric complications, which are detailed in Table 2. There were nine (2.7%) neonatal deaths including three that were not viable being less than 24 weeks gestation. PPH was the most common complication encountered following OOH birth. Two breech births occurred before the arrival of paramedics (Table 3).

**Table 2:** Complications encountered BBAs

Complication	Number	Incidence (%)
Breech presentation	4	1.3
Shoulder dystocia	2	0.6
Face Presentation	1	0.3
Cord prolapse	2	0.6
Twins	3	0.9
Primary PPH BBA	21	6.5
Incidence (out of 324 BBAs)	34	9.3

**Table 3:** Procedures performed on mothers by paramedics

Procedures	Number	Incidence (%)
Fundal massage	76	23.5
Methoxyflurane administration	98	30.2
Insertion of IV therapy	20	6.2
Oxygen	16	4.5
Cardiac monitor	9	2.7
IV Morphine	6	1.9
Auto infusion	3	0.9
IV Fentanyl	2	0.6
IV Metoclopramide	2	0.6
IV Prochlorperazine	1	0.3

As well as encountering birth complications, paramedics encountered at least 54 women (16.7%) who had other pre-existing medical and/or obstetric conditions that had the potential to complicate the births. During their history taking paramedics elicit information regarding previous medical conditions and current medications. However, all information is voluntarily provided by the women thus paramedics can only document the information they are given. Pre-existing medical conditions included asthma (*n* = 19), hypertension (*n* = 4), epilepsy (*n* = 3), hepatitis C (*n* = 3), multiple sclerosis (*n* = 1), diabetes mellitus Type 1 (n = 1) and Type 2 (*n* = 2). Conditions that commenced after pregnancy included gestational diabetes (*n* = 7) and pre-eclampsia (*n* = 5). A small number of women were previously diagnosed with psychiatric conditions (*n* = 10). Of all the women having unplanned OOH births, 2.7% (*n* = 9) were either taking illicit or prescription drugs which may have adversely affected their babies at birth including heroin (*n* = 4), methadone (*n* = 2) or both (*n* = 2). Furthermore, one fifth (*n* = 6, 20.7%) of the women who had premature babies with gestations less than 36 weeks, were using illicit drugs with one of these not viable at 20 weeks gestation. Paramedics did not document the gestation of the babies for two women.

### Management performed by paramedics at OOH births

Paramedics documented a variety of interventions including medication administration on both mother and baby (Tables 3 and 4). Procedures performed (Table 3) by paramedics on women included fundal massage (*n* = 76, 23.5%), oxygen administration (*n* = 16, 4.5%) and intravenous therapy (*n* = 20, 6.2%). Prior to birth, analgesia and antiemetic medication were administered to one third of women. Most of these received methoxyflurane (*n* = 98, 30.2%) with a small number also receiving an intravenous narcotic, either morphine (*n* = 6, 1.9%) or fentanyl (*n* = 2, 0.6%). Three women were also given antiemetic medication, either metoclopramide (0.6%, *n* = 2) or prochlorperazine (0.3%, *n* = 1).

**Table 4:** Procedures performed on babies by paramedics

Procedures	Number	Incidence (%)
Cut cord	186	57.4
Oxygen administration	62	19.1
Airway suction & Manual airway clearance	33	10.2
Intermittent Positive Pressure Ventilation	6	1.9
CPR	4	1.2
Adrenaline	2	0.6
Insertion of endotracheal Tube	1	0.3
Intraosseous fluid	1	0.3
Atropine	1	0.3

Documented procedures performed by paramedics on babies (Table 4) included cutting the umbilical cord (*n* = 186, 57.4%), oxygen administration (*n* = 62, 19.1%), airway management including suction and manual clearance (*n* = 33, 10.2%), intermittent positive pressure ventilation (*n* = 6, 1.9%). Paramedics documented that they performed full neonatal resuscitation on four babies (1.2%), including insertion of endotracheal tube for one baby (0.3%) by intensive care paramedics. In addition, they documented administering intraosseous fluids (*n* = 1, 0.3%) and medication during resuscitation including adrenaline (*n* = 2, 0.6%).

## Discussion

Overall, this study clearly demonstrates that paramedics are called to attend and provide care for OOH births. Almost 60% of births occurred prior to ambulance arrival including women birthing alone. Mothers were more likely to be multigravidas but paramedics did provide care for a small proportion of women having their first babies. There was a tendency for the women, who called paramedics for childbirth, to be from lower socioeconomic areas. The majority of the babies were born healthy at term and would be considered low risk in a planned birth setting. However, for a small proportion of the births, paramedics were required to manage premature babies or birth complications. Furthermore, paramedics encountered one in five women who had medical histories which could complicate their clinical management. One in 20 women admitted to taking either illicit or prescription drugs, with the potential to adversely affect their babies at birth. OOH births were more likely to occur outside business hours especially overnight.

For the year of the study paramedics responded to 462,830 emergency incidents [21], subsequently managing OOH births forms a small but significant portion of their workload. Additionally for the same year, the total number of births in Victoria was 71,586 with 300 unplanned out of hospital births officially documented [22] p.55. Although matching the cases from this dataset to published government perinatal statistics [22] was not possible in this study, it is likely paramedics attended the majority, if not all, unplanned out of hospital births recorded for that year.

Findings from this study largely support previous investigations into unplanned out of hospital births. Whilst the majority of OOH births are term babies, there is a greater possibility that these babies will be preterm compared to all in-hospital births [2, 4, 23]. Furthermore, this study reinforced previous research findings from outside Australia with the majority of the births occurring outside business hours [2, 4] and in the family home [4, 11, 23], regardless of the presence of paramedics or not. As with previous studies [11, 23], this research also highlighted a small percentage of women who had OOH births had little or no antenatal care, some with unknown pregnancies. Ten percent of women gave birth alone, usually at home. Likewise it further supports that OOH births have a greater incidence of perinatal deaths compared to in-hospital and planned home births [6]. These issues are of concern warranting further research.

Conversely, this study does differ from previous studies in some aspects. There was little evidence to support that OOH births are more likely to occur in rural locations due to travelling long distances or closure of smaller obstetric units [7]. Unlike earlier studies [9], this study did not find an increased risk of PPH associated with OOH births. Hence, this study can neither support nor refute previous assertions [24] that attendance of paramedics at OOH births has no effect on outcomes. Further investigation involving multivariate analysis is warranted. Neither can this study support nor refute previous findings [2] suggesting immigrants and non-English speaking are at greater risk of OOH births. Further research is required to explore these issues.

With limited resources to monitor progress during third stage, arrival following birth places paramedics in an extremely vulnerable position. As haemorrhage is a leading cause of maternal death during the immediate postpartum period [25], correct assessment and management of third stage of labour is critical. Paramedics reported 21 women had bleeding greater than 500mls defined as primary postpartum haemorrhage [26]. Whilst the incidence was on par with in-hospital births [27], it is reasonable to assume paramedics should have the knowledge and clinical judgement required to manage this potentially life-threatening situation. Whilst 21 women were documented to have PPH, ‘fundal massage’ was documented for 76 women, perhaps indicating either a lack of knowledge of the clinical indications for fundal massage or misunderstanding of the term ‘fundal massage’ [25].

Whether arriving before or after the birth of the baby, paramedics undoubtedly are required to utilise an extensive repertoire of assessment and clinical skills to prevent complications. For paramedics arriving before the birth, critical decision-making points in this process are sequential and involve both obstetric knowledge and paramedic competency; firstly, to establish whether there is time to transport to hospital or if the woman will birth imminently; then to assess at regular intervals, signs of impending birth which will impact on the continuation or temporary cessation of transportation [14].

Whilst acknowledging the majority of newborns were born at term with minimal complications, this study also demonstrates that paramedics are required to assess all newborn babies and perform a variety of interventions. Thirteen babies (4%) were recorded with APGAR scores less than three, with four babies (1.2%) receiving full neonatal resuscitation which is well above the national reported in-hospital rate [28]. Nearly one third of women were given analgesia including Methoxyflurane, Morphine and Fentanyl whilst in paramedics’ care, which are not recommended immediately prior to the birth of a baby due to increased risk of neonatal respiratory depression [29, 30]. In the immediate period after birth paramedics suctioned the airways of and administered oxygen to more babies than required resuscitation, again highlighting the need for paramedics to keep abreast of current evidence underpinning neonatal resuscitation [31, 32]. With nine neonatal deaths, paramedics should also be cognisant of accepted approaches to provide emotional support to parents for perinatal deaths [33].

This study demonstrates the importance of educating paramedics about the changes immediately after birth for both mother and baby to effectively care for them during this period. The importance of being be able to recognise the signs of imminent birth and make decisions accordingly highlights the need for paramedics to be aware of current practices for women in second stage. Simultaneously they must ensure both the baby is adapting to the extra-uterine environment and that third stage is progressing with no complications, requiring considerable knowledge. Their clinical skills must enable them to monitor the progress of both newborn and mother, responding early to any complications that may arise.

Conversely, most of the women encountered had uncomplicated term pregnancies so paramedics should be taught the importance of normalising these births. Having the theoretical knowledge that underpins practice cannot be underestimated. Learning the expected maternal physiological changes and the normal transition of the newborn after birth should be embedded in paramedics’ curriculum. In turn, paramedics should be familiar with normal practices following birth such as the importance of early skin-to-skin [32, 34], delayed cord clamping [32, 35] and promoting early breast feeding [36]. Giving birth can be a dynamic period and the presence of paramedics supporting women with either complicated or uncomplicated births confirms the need for them to develop proficient clinical skills.

As highly skilled emergency care practitioners, paramedics are required to manage people in a range of health conditions including birth. Although a normal process, birth is an important event for women and their families. This could be particularly true when the birth of a baby occurs in unexpected locations, placing paramedics in a potentially vulnerable position. Whilst there are no current publications investigating litigation against any Australian ambulance services, the London Ambulance service has reported the cost of mismanaged obstetric cases is reportedly disproportional to the small number of actual cases managed [37]. The general public expects paramedics to be up-to-date with managing OOH births [38]. This range of events managed by paramedics during birth highlights the need for comprehensive education and certification programmes. Australian paramedics are not currently registered health professionals, so no external body regulates their education and ongoing competence unlike their United Kingdom counterparts [39].

This study demonstrates that paramedics attend, support and provide treatment for women having OOH births in the community. However, this remains an under-researched area requiring further investigation. Whilst birth is a small, albeit important, part of the childbearing experience, little is known about the types of cases paramedics attend during pregnancy and the postpartum period. Until there is a clearer picture of the maternity emergency care performed by paramedics, it is difficult to judge their total educational requirements in this area and the support they require when making clinical and transport decisions.

### Limitations

While this study highlights the important role that paramedics play during OOH births, several limitations are acknowledged. The study focuses on care provided by a single state-wide ambulance service, hence whilst similarities to other states, territories and countries may occur, generalisations to other jurisdictions cannot be drawn. This was a retrospective study and data entry was completed by paramedics in the field for the purposes of clinical management, often in stressful situations, and resulting in variation in the amounts and type of documentation recorded. As the electronic information system used by paramedics must be able to capture a large amount of information about wide variety of health conditions, there were limited specific obstetric variables available to prompt paramedics to document all relevant data. While free text allowed paramedics to document information that could not be entered into existing fields, the content was dependent upon the knowledge of the treating paramedic. For this reason, the data in this study was inconsistent and incomplete. The creation of core minimum data sets for labouring and birthing women is recommended.

The data in this study was extracted from a population-based clinical database designed for purposes other than research. To ensure this data would reflect a clinical picture as close to reality as possible, the data were meticulously recoded but has resulted in reporting data that could be considered dated. Whilst, recoding of the data proved extremely time consuming, it also makes this study relevant and unique. Consistent monitoring of ambulance clinical records to identify women with maternity-related conditions would ensure up-to-date data could easily be retrieved for audits and research.

At least one in seven women attended sought advice from the hospital during labour prior to birth. Previous studies [1, 8] have suggested that encouraging labouring women to stay home longer before going to the birthing suite could contribute to the possibility of unplanned out of hospital births. On the other hand, there is strong evidence to suggest that women who go to hospital too soon have greater risk of the cascade of interventions and caesarean sections [40–42]. Whilst this study is limited in drawing any conclusions, it highlights the need for further research regarding the appropriate time for women to go to hospital and recommended strategies for managing precipitous labour.

Victorian paramedics do not collect specific demographic information relating to country of birth or socioeconomic status. Although previous studies report that women experiencing OOH births are more likely from lower socioeconomic areas [2], substance abusers [2] or from non-English speaking backgrounds [2], this study provided only limited insight into these assertions. In addition, Victorian paramedics do not have neonatal thermometers available making assessing the baby’s body temperature difficult, so this study could neither support nor refute increased risk of hypothermia for OOH births found in other previous studies [1, 8].

## Conclusion

Paramedics provide emergency health care for pre-hospital birthing women. Although the majority of births were uneventful precipitous births at term requiring minimal intervention, OOH births present high-risk clinical challenges. For a small proportion of births the risk is compounded due to emergencies including prematurity, maternal factors and obstetric complications. Regardless of degree of complications encountered, paramedics perform complex obstetric assessments, judgements and clinical skills regarding progress and transportation of these women. The findings of this study are valuable in that they provide important information that has not previously been available. Implications of these findings impact upon paramedic clinical practice and education for management of out-of-hospital emergency births.

## Abbreviations

BBA: An unexpected birth that occurs in either an unplanned location or without the pre-organised birth attendant such as home birth midwife
OOH births: Out of hospital births which is another term for BBA
PPH: Primary Postpartum Haemorrhage
